# Consumer Perceptions of the Canadian Salmon Sector and Their Associations with Behaviors: A Perspective from Indigenous Rights

**DOI:** 10.3390/foods13091309

**Published:** 2024-04-24

**Authors:** Sylvain Charlebois, Ning Sun, Ken Paul, Isaiah Robinson, Stefanie M. Colombo, Janet Music, Swati Saxena, Keshava Pallavi Gone, Janele Vezeau

**Affiliations:** 1 Agri-Food Analytics Lab, Dalhousie University, Halifax, NS B3H 4R2, Canada; sylvain.charlebois@dal.ca (S.C.); janet.music@dal.ca (J.M.); 2 Wolastoqey Nation at Neqotkuk, Rowena, ON E7H 5M8, Canada; ken.paul@oceanfi.ca; 3 Kitasoo Xai’xais Nation, Klemtu, BC V0T 1L0, Canada; deputy@kitasooband.com; 4 Faculty of Agriculture, Dalhousie University, Truro, NS B2N 5E3, Canada; scolombo@dal.ca; 5 Department of Biology, University of Toronto, Toronto, ON M5S 1A1, Canada; swati90lko@gmail.com; 6 Faculty of Management, Dalhousie University, Halifax, NS B3H 4R2, Canada; keshavapallavi.gone@dal.ca; 7 Canadian Agri-Food Foresight Institute, Halifax, NS B2X 3T5, Canada; janele.vezeau@cafi-icpa.ca

**Keywords:** farmed salmon, consumer behavior, environmental sustainability, economic considerations, indigenous rights

## Abstract

Previous studies on consumer perceptions and behaviors of salmon have often neglected Indigenous rights within the Canadian salmon sector. This study innovatively addresses this gap by integrating Indigenous rights into the current analysis, alongside considerations of sustainability practices, socio-economic impacts, and consumer motivations. Our research objectives aim to fit three consumer perceptions—environmental sustainability, economic considerations, and Indigenous rights—and to evaluate their associations, alongside perception of a price increase, socio-demographics, and consumer motivation factors, with purchasing behaviors related to Canadian salmon products. Data for this study was collected from a nationwide online survey. Responses to Question 2 and Question 35 are encoded with numerical values ranging from 1 to 5, where larger numbers indicate stronger agreement with the statement. The inclusion of methodologies such as the Graded Response Model (GRM) and Cumulative Link Models (CLM) adds another innovative dimension to this study. Our findings demonstrate how consumer profiles are associated with these four perceptions and their underlying determinants. Furthermore, the study quantifies the influence of these four perceptions on each consumer purchase behavior. The implications of these findings extend to the realm of mathematical modeling in consumer decision-making processes, offering practical insights for businesses and marketers, and emphasizing the importance of implementing regulatory frameworks and initiatives that promote sustainability, safeguard Indigenous rights, and address socio-economic disparities.

## 1. Introduction

Farmed salmon constitutes Canada’s third-largest seafood export by value, accounting for over 70% of the total production volume and more than 80% of the overall farm-gate value. While salmon cultivation occurs in Atlantic Canada, the primary hub of the industry is in British Columbia, the largest agri-food export region. Salmon aquaculture in British Columbia commenced in the late 1970s, and by 2021, the industry produced 84,171 metric tonnes with an economic value of CAD 692,381,000. The salmon farming sector has played a pivotal role in managing freshwater and marine resources, contributing significantly to the economic and social well-being of coastal communities [1].

Salmon consumption in Canada exhibits a remarkable degree of prevalence, as shown by empirical studies indicating that approximately 79% of the population integrate this fish into their dietary habits, with 10% consuming salmon weekly [2]. These dietary habits are likely influenced by factors such as sustainable development, economic considerations, and cultural habits. Primarily, a survey revealed strong support for sustainable practices in Canadian salmon farming. Over half (55%) of Canadians expressed a purchase intent for farmed salmon raised on environmentally friendly and nutritious diets, and 54% believed that aquaculture is a sustainable method for salmon harvesting in Canada [2]. These findings suggest broad public approval for the sustainability of ocean farm production. Moreover, economic considerations such as employment, income, and the potential for severe declines or loss of economic benefits from harvest are taken into account by Canadians [3]. Beyond these factors, salmon consumption plays a crucial role in fostering cultural ties among First Nations communities. Traditional practices of food-sharing and communal feasting centered around salmon serve to bolster cultural identity, social connectedness, and cultural continuity—all of which are positively associated with the health and well-being of these communities [4].

Research on salmon has consistently been a focal point in the domain of consumer perception analysis. Osmond et al. conducted an exploratory investigation into Canadian consumer perceptions and behaviors regarding salmon [2]. Zheng et al. employed a Random Parameters Logit (RRL) model to scrutinize heterogeneity in preferences, integrating perceptions of genetically modified (GM) farmed salmon [5]. Grundvåg Ottesen provided valuable insights into Norwegian consumer perceptions of salmon [6], while Gaedeke explored perceptions regarding both wild-caught and farmed salmon [7]. Numerous studies have delved into the influence of factors on consumer perceptions and behavior. Qin and Brown investigated the impact of process and product-related information [8], Budhathoki et al. delved into the place of purchase and provision of production method [9], Nickoloff et al. explored the effects of conflicting health information [10], Onozaka et al. focused on geographic origin coupled with sustainable measures [11], Whitmarsh and Palmieri examined environmental preferences [12], and Muñoz-Colmenero et al. identified economic reasons favoring wild-caught over farmed products as primary drivers [13]. Moreover, research has explored the intricate relationship between perception and behavior. Onozaka et al. employed Latent Class Analysis (LCA) embedded in Structural Equation Modeling (SEM) to study the impact of perceptions of healthiness, value for money, and convenience on salmon consumption frequencies [14]. Zheng et al. utilized an ordered logit model to investigate perceptions of consumption attributes (clean, tasty, nutritious) and their influence on purchase habits of Alaskan salmon [15]. Suzuki et al. employed SEM to explore the post-disaster consumer perception effect on seafood purchase intent [16]. Zheng et al. applied the RPL model to assess Chinese consumers’ willingness to pay, incorporating perceptions of the production environment (wild-caught and farm-raised) and food safety attributes [17]. Alfnes et al. designed 20 choice scenarios with posted prices to investigate consumers’ willingness to pay for the color of salmon using a mixed logit model [18]. Myrland et al. applied the Fishbein–Ajzen approach to study consumer perception and the frequency of Norwegian salmon consumption [19]. Despite the limitations observed in these studies, such as the restriction of analytical variables to singular questions in their surveys, resulting in potential subconscious biases among respondents, their significance for the advancement of the Canadian salmon sector persists. For instance, initiatives like the enactment of the Fisheries Act and the management of farm salmon licenses have been guided by imperatives of sustainable practices, economic considerations, consumer motivations (such as price, origin, attributes, and consumption frequencies), and cultural habits.

However, in early 2023, the Canadian government chose not to renew licenses for 15 salmon farms around British Columbia’s Discovery Islands, following a prior decrease in license issuances by 22 in 2022 [20]. This policy shift within the salmon farming industry resulted in an approximate 25% reduction in the total number of salmon farms in British Columbia [21]. This governmental intervention carries noteworthy implications. Primarily, it is poised to create a scarcity of Canadian salmon, leading to a 13% reduction in exports and a decline in Canada’s market share within the US market. Furthermore, this regulatory measure is anticipated to trigger a 12.7% increase in the retail price of Canadian salmon [21], potentially prompting Canadian consumers to substitute domestically farmed salmon with imports or other seafood. Considering that salmon holds the first position in Canada’s seafood choices [22], such a shift may adversely impact Canadians’ consumption satisfaction. Additionally, the subsequent exacerbation of potential adverse impacts leads to changes in employment and income levels in Canadian coastal and Indigenous communities, many of which have faced economic challenges stemming from declines in resource-based industries [23]. Specifically, the recent shutdown order is estimated to result in the loss of over 300 jobs for Mowi workers [24].

Salmon has also been intrinsically linked to Indigenous livelihood and culture for centuries. Notably, the historical entwinement of Alaska Natives with salmon extends beyond 10,000 years [25]. However, recent decades have borne witness to a decline in wild salmon populations attributed to multifaceted factors, including climate change, overfishing, and diseases [26]. While the Fisheries Act prioritizes Indigenous ceremonial and subsistence fishing, the loss of cultural diversity has not received the same attention as the loss of biological diversity [27]. Research conducted by Carothers et al. highlighted the impending threats to the sustainability of six Sugpiaq villages in the Kodiak Archipelago due to lost fisheries access and the cumulative impacts of restricted access management [25]. Similarly, Steel et al. underscored the mounting challenges faced by Haíłzaqv Nation fishers in accessing salmon within their traditional territories due to escalating fuel costs and boat maintenance fees [28].

The controversy surrounding the decrease in the issuance of salmon farm licenses in British Columbia reflects either the potential neglect of critical factors in previous research within the Canadian salmon sector or the presence of unresolved limitations in existing studies, both of which exert ongoing influence on the sustainability and economic management policies of the current government. Building upon insights derived from previous research, this study innovatively addresses this gap by integrating Indigenous rights into the current analysis of consumer perceptions and behaviors, alongside considerations of sustainability practices, socio-economic impacts, and consumer motivations. Our research objectives aim to fit three consumer perceptions—environmental sustainability, economic considerations, and Indigenous rights—and to evaluate their associations, alongside the perception of a price increase, socio-demographics, and consumer motivation factors (with specific emphasis on “the perceived importance of price” and “the perceived importance of origin”), with each purchasing behavior related to Canadian salmon products. Moreover, the incorporation of methodologies such as the Graded Response Model (GRM) and Cumulative Link Models (CLM) [29] adds another innovative dimension to this study. The GRM is applied to capture latent traits underlying perceptions within each sub-domain, while Cumulative Link Models are employed to conduct analyses of consumer perceptions and behaviors. A nationwide online survey was also conducted.

This study is strategically positioned to provide valuable insights for both policymakers and stakeholders within the salmon industry. By leveraging the findings of this research, policymakers can effectively navigate the complex challenge of balancing environmental sustainability with Indigenous rights in the formulation of salmon farm licensing policies. This sophisticated approach could involve the development of policies that endorse a sustainable number of licenses for farmed salmon production, not only catering to established consumer demand but also incorporating culturally sustainable measures to support the livelihoods and employment opportunities of Indigenous communities, including Indigenous-led programs. Given its significance as an economic driver for Canadian aquaculture and seafood production [23], this balanced approach is crucial for bolstering the salmon sector’s contribution to Canada’s Blue Economy Strategy. Stakeholders, in turn, will gain a comprehensive understanding of purchasing behaviors aligned with consumer perceptions, enabling informed adjustments to enhance market strategies accordingly.

The rest of the study is organized as follows. Section 2 explains the theoretical framework. Section 3 describes data collection procedures, descriptive data analysis, and the statistical models used in the research. In Section 4, a three-step procedure is employed to fit three perceptions, examining their correlations with socio-demographic variables and consumer motivations, followed by assessing the associations with seven purchasing behaviors, respectively, through CLM. Section 5 initiates a discussion on the findings, comparisons, limitations, and future work. Finally, Section 6 concludes the study.

## 2. Theoretical Framework

### 2.1. Bias Reduction in Survey Design

Subconscious bias [30] represents a prevalent limitation in survey studies. Concerning academic concepts such as sustainability, economics, and Indigenous rights, respondents may harbor diverse interpretations, predisposing their responses to subconscious biases. Moreover, ensuring consistency across responses within related topical questions (referred to as sub-domains herein) poses a challenge in the Likert scale surveys. Respondents may encounter difficulty in consistently aligning their responses, particularly when faced with choices ranging from agree/disagree to strongly agree/disagree, as well as neutral. Such variations in responses inevitably impact the final analytical outcomes of the study. To reduce this subconscious bias, we structure subsections of questions regarding targeted perceptions in the questionnaire, avoiding reliance on singular questions for depicting a targeted perception. This design acknowledges that individuals may not be able to accurately capture their true perception in a single depicted question. By using a dimension technique in each sub-domain, we anticipate obtaining a more accurate estimation of the targeted perception of respondents, thus also mitigating potential deviations stemming from the Likert scale choices regarding targeted perception.

### 2.2. Study Framework

Our survey is informed by the conceptual framework proposed by Charlebois et al., which incorporates consumer motivations, sustainability, and economic dynamics [31]. This survey is divided into several subsections: agreements, socio-demographics, consumer motivations, sustainability, and economic considerations. Questions within each subsection are formulated drawing inspiration from the work of Osmond et al. [2], for instance, ‘I would prefer to buy organic salmon over regular farmed salmon’. Related perceptions and behaviors are addressed in each subsection. In particular, perceptions regarding Indigenous rights are included in these subsections. This design is aimed at mitigating potential selection bias among respondents and facilitating the objective capture of their perceptions.

**Figure 1:**
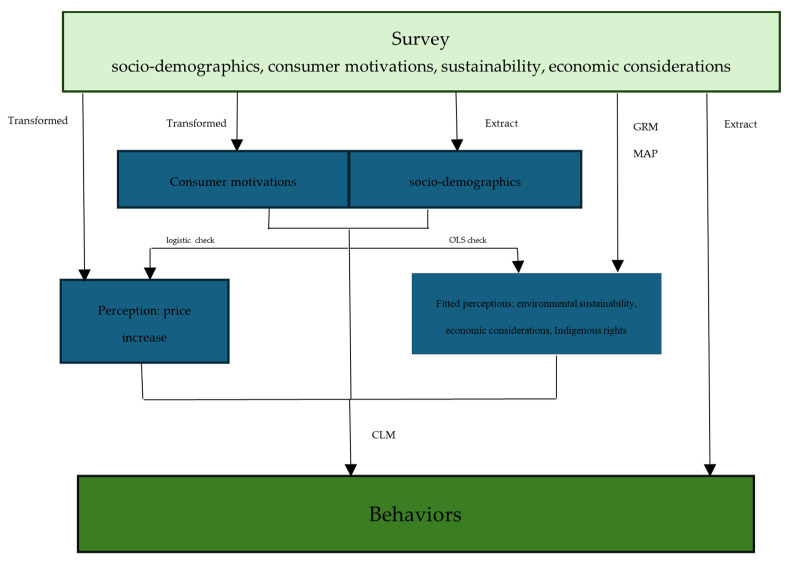
The flow chart of the procedure of this study.

The procedure of our study is illustrated in Figure 1. Our main objective is to explore the associations between perceptions and behaviors within the Canadian salmon sector, while also examining consumer motivations (price and origin importance) and socio-demographics. The motivational factors are informed by the studies of Alfnes et al. [18] and Onozaka et al. [11], whereas the perception of a price increase is influenced by research from CAIA [21] and Zurek and Rudy [32] following the COVID-19 pandemic. The adjustment of these three variables is necessitated due to their Likert scale measurement, where distinctions between moderate and strong importance/increase are not essential, resulting in the derivation of three new binary variables.

The second crucial step involves fitting three perceptions within each sub-domain (environmental sustainability, economic considerations, and Indigenous rights), which are selected from all related questions in the survey. This necessitates conducting dimensionality tests. Various dimension techniques are available, including factor analysis [16]. In our study, we employ parallel analysis to determine the dimensionality d within each sub-domain. Subsequently, the Graded Response Model (GRM) and Maximum A Posteriori (MAP) techniques are applied for estimating perceptions in each sub-domain. A comprehensive explanation of these methodologies is presented in Section 4.

In the third stage of the study, ordinary least squares (OLS) and logistic regression models are employed to investigate the relationships between four perceptions, consumer motivations, and socio-demographics. Lastly, Cumulative Link Models (CLMs) are utilized to fulfill our main research objective.

## 3. Methodology

### 3.1. Data Collection

Data collection for this study was conducted through a nationwide online survey administered from 8 January to 15 January 2024. The survey employed a sample size of 1101 Canadian residents, selected using a randomized approach within regions of Canada, including British Columbia, the Prairies, Ontario, Quebec, and the Atlantic provinces. Angus Reid was entrusted with survey administration to facilitate the random selection of participants. To ensure comprehensive representation across age and gender categories within each region, the determination of the sample for each was guided by the imperative to align with the data guidelines established by Statistics Canada in 2020. Participation in the survey was entirely voluntary, and individuals who chose to participate received an electronic message containing a comprehensive survey description along with the link to access it. Throughout the entire survey process, the anonymity of participants was maintained.

Before the official commencement of the online survey, a translation of all questions into French was undertaken, accompanied by subsequent adjustments and verification. The questionnaire encompasses 2 items featuring agreement-based questions, 1 open-ended question, 10 socio-demographic queries, and 34 Likert scale questions. For this study, exclusive focus is given to closed questions. A copy of the questionnaire is appended in File S1 of the Supplementary Material.

### 3.2. Descriptive Statistics

A total of 1101 independent observations were initially obtained through the survey. Question 1 is designed to determine eligibility, requiring participants to confirm that they were over 18 years old and had resided in Canada for the last 12 months. Question 2 aims to assess participants’ understanding of the survey. Responses marked as ‘disagree’ in either Question 1 or Question 2 were excluded, along with one instance of missing data in a Likert scale question. This resulted in a final sample size of 1078. Notably, the category ‘Raw foodist (a diet consisting mainly of raw fruits, vegetables, legumes, sprouts, and nuts)’ was consolidated with the ‘Other’ category due to the presence of only one observation. Frequencies for socio-demographics are detailed in Table 1.

We also have encoded the 34 Likert questions from Q2 to Q35 as follows: ‘1’ for ‘strongly disagree’, ‘2’ for ‘disagree’, ‘3’ for ‘neither agree nor disagree’, ‘4’ for ‘agree’, and ‘5’ for ‘strongly agree’. Table 2 provides frequencies for these 34 variables.

### 3.3. Statistic Models

#### 3.3.1. Graded Response Model

When each increasing option of questions reflects increasing levels of the perception domain, the Graded Response Model (GRM) [33] can be used to summarize all questions in the domain. Suppose a perception domain has J items with each item j has possible options $1,2,\;\ldots ,K_{j}$ for j=1, 2…, J. Then the GRM estimate the probabilities of answering $k\in \left\{1,\;2\ldots ,K_{j}\right\}$ through $P_{k}\left(\theta \right)=P\left(Y_{j}=k|\theta \right)=P\left(\alpha_{\left(j,k-1\right)}<Z_{j}\leq \alpha_{\left(j,k\right)}|\theta \right),$ where $Y_{j}$ represents the answer of the respondent, θ is a d-dimensional latent trait used to characterize the domain, $Z_{j}$ is a latent continuous variable for item j and $\alpha_{\left(j,k-1\right)},\alpha_{\left(j,k\right)}$ are the corresponding threshold parameters to be estimated. The relationship between the latent trait θ and the latent variable $Z_{j}$ can be written as Z=Λθ+ϵ where $Z={\left[Z_{1},\ldots ,Z_{J}\right]}^{T}$, Λ is the slope matrix with J rows and d columns, and ϵ is a J-dimensional Gaussian random vector.

Given the observations ${\left\{Y_{i1},\ldots ,Y_{iJ}\right\}}_{i=1}^{n}$ for the perception domain, the GRM estimates $P_{k}\left(\theta \right)$ for each item j=1, 2…, J by estimating the slope matrix Λ and the thresholds parameters ${\left\{\alpha_{\left(j,k\right)}\right\}}_{1\leq j\leq J,0\leq k\leq K_{j}}$. The resulting curves of $P_{k}\left(\theta \right)$ for $k\in \left\{1,\;2\ldots ,K_{j}\right\}$ are called trace plots of item j, indicating how the probability of responding k to item j will change according to the change of the latent trait θ. Once slope matrix Λ and the thresholds parameters ${\left\{\alpha_{\left(j,k\right)}\right\}}_{1\leq j\leq J,0\leq k\leq K_{j}}$ are estimated, the maximum a posteriori (MAP) estimator of latent trait ${\hat{\theta }}_{i}$ can be obtained by maximizing the posterior distribution of $\theta_{i}$ given $\hat{\Lambda }$ and ${\left\{{\hat{\alpha }}_{\left(j,k\right)}\right\}}_{1\leq j\leq J,0\leq k\leq K}$ for each participant i=1,…, n. The resulting MAP estimator ${\hat{\theta }}_{i}$ of $\theta_{i}$ is defined as the measurement of the level of the perception domain in this paper.

#### 3.3.2. Multiple Linear Regression

If the latent trait θ is a scalar, i.e., d=1, then it can be used as a continuous outcome for a multiple linear regression model and fitted by the ordinary least squares (OLS) estimation. The variables of the perceived importance of price (Q13) and the perceived importance of origin (Q14) are dichotomized by combining the answers ‘Strongly disagree,’ ‘Disagree’, and ‘Neither agree nor disagree’ into the category ‘Do not perceive as important’, while the answers ‘Agree’ and ‘Strongly agree’ are combined into the category ‘Perceive as important’. The new transformations are defined as $\tilde{Q13}$ and $\tilde{Q14}$. To study the relationship between perception domains and consumer profiles, the following regression model is estimated: $\theta_{i}=\beta +{sociodemographic}_{i}+{\tilde{Q13}}_{i}+{\tilde{Q14}}_{i}+\varepsilon_{i}$, where β is the intercept. The socio-demographic variables (question D1–D9 and Q1) are all transformed into binary dummy variables.

#### 3.3.3. Logistic Regression

The perception of a price increase (Q34) is dichotomized by combining the answers ‘Strongly disagree’, ‘Disagree’, and ‘Neither agree nor disagree’ into the category ‘Do not perceive a price increase’, while the answers ‘Agree’ and ‘Strongly agree’ are combined into the category ‘Perceive a price increase’. The new transformation is defined as $\tilde{Q34}$. Hence, the percentage of a perceived price increase can be analyzed using logistic regression: $\log\left(\frac{p}{1-p}\right)=\beta +{sociodemographic}_{i}+{\tilde{Q13}}_{i}+{\tilde{Q14}}_{i}$, where β is the intercept. The socio-demographic variables (questions D1–D9 and Q1) are all transformed into binary dummy variables.

#### 3.3.4. Cumulative Link Model

The consumer purchasing behavior is analyzed using the cumulative link model (CLM) [29] with the logistic link function:

$$\log\left(\frac{P\left(Y_{i}\leq j\right)}{1-P\left(Y_{i}\leq j\right)}\right)=\gamma_{j}-{sociodemographic}_{i}-\theta_{i}^{\mathrm{Sustainability}}-\theta_{i}^{\mathrm{Economics}}-\theta_{i}^{\mathrm{Indigenous}}-{\tilde{price\;increase}}_{i},\;j=1,2,3,4$$

where $Y_{i}$ is respondent i’s answer to the question related to consumer purchasing behavior, $\gamma_{j}$ is the threshold parameter, $\theta_{i}^{\mathrm{Sustainability}}$, $\theta_{i}^{\mathrm{Economics}}$, and $\theta_{i}^{\mathrm{Indigenous}}$ are the latent traits of environmental sustainability, economic considerations, and Indigenous rights obtained by the GRM. All analyses are conducted using R (version 4.3.2).

## 4. Results

### 4.1. Cronbach’s Alpha Reliability Test

The Likert scale questions demonstrate a robust level of internal consistency, as shown by Cronbach’s alpha coefficients of 0.921, 0.852, 0.795, and 0.923 in Table 3 for the whole survey and each sub-domain (environmental sustainability, economic considerations, and Indigenous rights). Surpassing the commonly accepted threshold of 0.7 for internal consistency, these obtained values (0.921, 0.852, 0.795, 0.923) indicate a notably high degree of reliability in this survey.

### 4.2. Dimensionality Test: Parallel Analysis

The parallel analysis is used to determine d, the dimension of the latent traits θ for each sub-domain. The parallel analysis generates simulated random matrixes for obtaining the eigenvalues from the principal component analysis (PCA). The lowest component number for which the minimal PCA eigenvalue from the actual data is below that of the simulated data, is chosen as the value of d [34].

**Figure 2:**
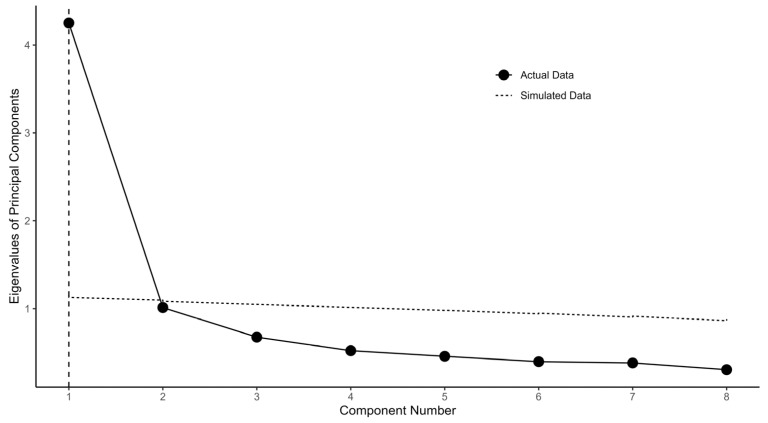
PCA eigenvalues for actual data (Q19–Q20 and Q22–Q27) and simulated data for parallel analysis.

Figure 2 depicts the comparison between the PCA eigenvalues from actual data of Q19–Q20 and Q22–Q27 (environmental sustainability domain) and randomly simulated data. Please note that the cross (eigenvalues of PCA from the actual data) stays below both the dotted (simulated data) and dashed (resampled data) lines for component numbers 2–8. This indicates d=1 for the environmental sustainability domain.

**Figure 3:**
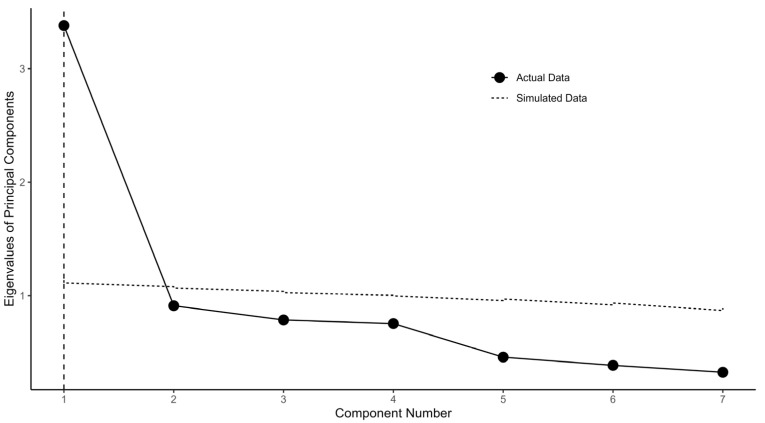
PCA eigenvalues for actual data (Q28–Q33 and Q35) and simulated data for parallel analysis.

Figure 3 depicts the comparison between the PCA eigenvalues from actual data of Q28–Q33 and Q35 (economical consideration domain) and randomly simulated data. Please note that the cross (eigenvalues of PCA from the actual data) stays below both the dotted (simulated data) and dashed (resampled data) lines for component numbers 2–7. This indicates d=1 for the economical consideration domain.

**Figure 4:**
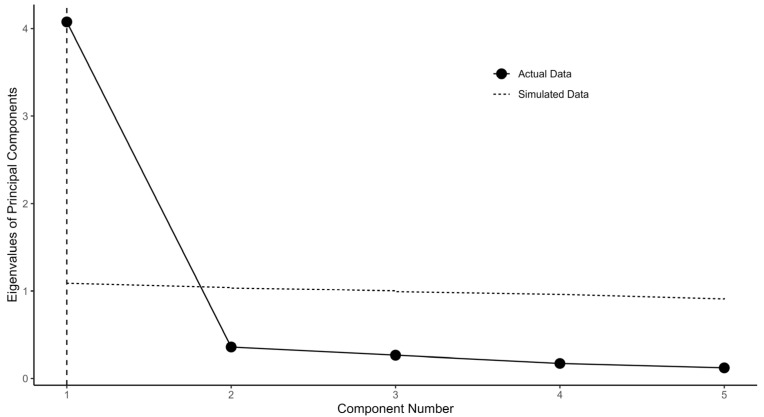
PCA eigenvalues for actual data (Q11–Q12, Q17, Q24, and Q29) and simulated data for parallel analysis.

Figure 4 depicts the comparison between the PCA eigenvalues from actual data of Q11–Q12, Q17, Q24, and Q29 (Indigenous rights domain) and randomly simulated data. Please note that the cross (eigenvalues of PCA from the actual data) stays below both the dotted (simulated data) and dashed (resampled data) lines for component numbers 2–5. This indicates d=1 for the Indigenous rights domain.

In sum, the dimension d=1 is confirmed by the parallel analysis for all three perception domains environmental sustainability, economic consideration, and Indigenous rights. This result simplifies the GRM model to the normal ogive model first discussed by Samejima [35].

### 4.3. The GRM Results

**Figure 5:**
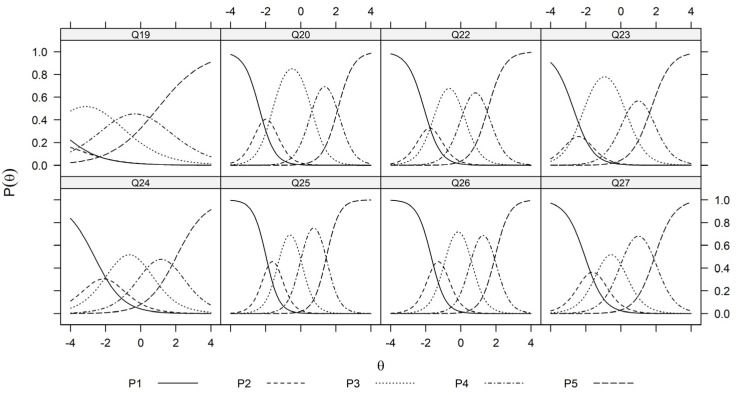
Trace plots of sustainability items (Q19, Q20, Q22, Q23, Q24, Q25, Q26, and Q27). θ represents the unidimensional latent trait (sustainability) and $P\left(\theta \right)\in \left\{P_{1}\left(\theta \right),P_{2}\left(\theta \right),P_{3}\left(\theta \right),P_{4}\left(\theta \right),P_{5}\left(\theta \right)\right\}$ represent the fitted probability of responding $k\in \left\{1,2,3,4,5\right\}$ to each of the question items Q19, Q20, Q22, Q23, Q24, Q25, Q26, and Q27 at the sustainability level θ.

As a unidimensional graded response model is recommended for developing the latent trait for items Q19, Q20, Q22, Q23, Q24, Q25, Q26, and Q27, for which ‘5’ represents the positive impact of salmon farming on environmental sustainability, the fitted probabilities for answering 1–5 given the latent trait $\theta^{\mathrm{Sustainability}}$ is depicted in Figure 5.

**Figure 6:**
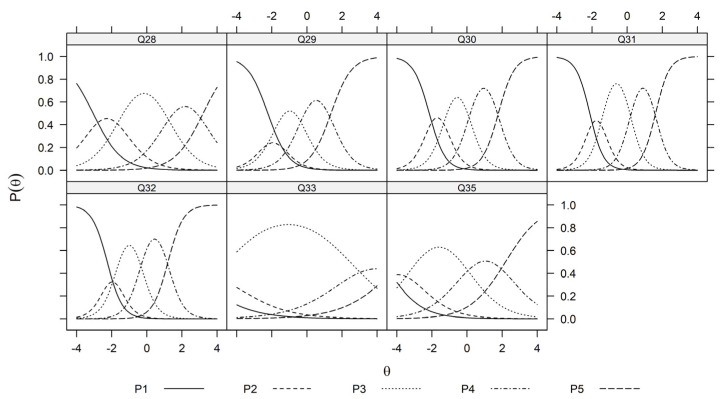
Trace plots of economic consideration items (Q28, Q29, Q30, Q31, Q32, Q33, and Q35). θ represents the unidimensional latent trait (economic consideration) and $P\left(\theta \right)\in \left\{P_{1}\left(\theta \right),P_{2}\left(\theta \right),P_{3}\left(\theta \right),P_{4}\left(\theta \right),P_{5}\left(\theta \right)\right\}$ represent the fitted probability of responding $k\in \left\{1,2,3,4,5\right\}$ to each of the question items Q28, Q29, Q30, Q31, Q32, Q33, and Q35 at the economic consideration level θ.

Similarly, the recommended unidimensional graded response model by the parallel analysis develops the latent trait for items Q19, Q20, Q22, Q23, Q24, Q25, Q26, and Q27, for which ‘5’ represent the positive impact of salmon farming on economic consideration, the fitted probabilities for answering 1–5 given the latent trait $\theta^{\mathrm{Economics}}$ is depicted in Figure 6.

**Figure 7:**
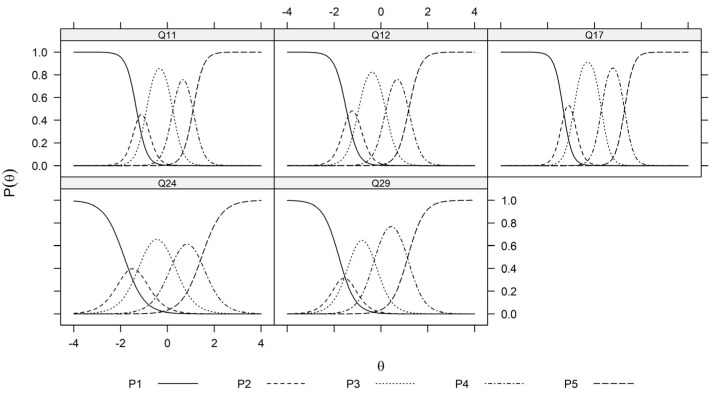
Trace plots of economic consideration items (Q11, Q12, Q17, Q24, and Q29). θ represents the unidimensional latent trait (supporting Indigenous communities) and $P\left(\theta \right)\in \left\{P_{1}\left(\theta \right),P_{2}\left(\theta \right),P_{3}\left(\theta \right),P_{4}\left(\theta \right),P_{5}\left(\theta \right)\right\}$ represent the fitted probability of responding $k\in \left\{1,2,3,4,5\right\}$ to each of the question items Q11, Q12, Q17, Q24, and Q29 at the level θ of supporting Indigenous rights.

Again, the recommended unidimensional graded response model by the parallel analysis develops the latent trait for items Q11, Q12, Q17, Q24, and Q29, for which ‘5’ represents the positive impact of salmon farming on supporting Indigenous rights, the fitted probabilities for answering 1–5 given the latent trait $\theta^{\mathrm{Indigenous}}$ is depicted in Figure 7.

Please note that the trace plots in Figure 5, Figure 6 and Figure 7 are based on the estimated values $\hat{\Lambda }$ and ${\left\{{\hat{\alpha }}_{\left(j,k\right)}\right\}}_{1\leq j\leq J,0\leq k\leq K}$. Hence by assuming standard normal priors, the MAP estimation of the latent traits can be obtained for all participants. The resulting values for each perception domain are summarized in Table 4.

Throughout the rest of the paper, we shall refer to ‘environmental sustainability’ as the MAP estimator of the latent trait $\theta_{i}^{\mathrm{Sustainability}}$, and ‘economic considerations’ as the MAP estimator of latent trait $\theta_{i}^{\mathrm{Economics}}$, and ‘Indigenous rights’ as the MAP estimator of latent trait $\theta_{i}^{\mathrm{Indigenous}}$ for all respondent i=1,…, n.

### 4.4. Perceptions and Consumer Profiles

Regression analysis on socio-demographic variables (D1–D9 and Q1), $\tilde{Q13}$ and $\tilde{Q14}$ is employed to examine their associations with fitted perceptions concerning environmental sustainability, economic considerations, Indigenous rights, and perception of a price increase. In this subsection, we scrutinize the variations in these perceptions across diverse consumer profiles. The findings are presented in Table 5.

The results elucidated in Table 5 unveil the negative associations of dietary preferences (particularly among respondents aligning with categories such as flexitarian, vegan, and others), gender (especially in the cases where respondents prefer to withhold disclosure), age, education (specifically those holding a registered apprenticeship or other trades certificate or diploma), and geographic location, specifically focusing on residency within British Columbia, with the perception of environmental sustainability in salmon farming. In contrast, household size, especially those characterized by a single child, and respondents residing in small towns, communities, or rural areas, along with the perceived importance of price and origin, demonstrate conspicuous positive significance in their associations with the perception of environmental sustainability in salmon farming.

Table 5 provides additional insights, revealing that dietary preferences (specifically aligning with categories such as flexitarian, vegan, vegetarian, and others), gender (particularly pronounced among non-binary/third-gender respondents and those preferring to withhold disclosure), age (notably within the range of the 1965–1979 bracket), education (especially the possession of a registered apprenticeship or other trades certificate or diploma), and geographic location (specifically for those residing in British Columbia) exert significant negative associations with the perception of economic considerations. Conversely, household size, especially those with one or two children, geographical location (with respondents residing in Quebec), residential zones (particularly those in urban cores), and the perceived importance of price and origin wield positive associations with the perception of economic considerations.

Table 5 highlights factors negatively associated with the perception of Indigenous rights. These include vegan and other dietary preferences, gender (especially for those who withhold disclosure), age (particularly those born before 1979), education (specifically the possession of a registered apprenticeship or other trades certificate or diploma), and geographic location (specifically for those residing in British Columbia). Conversely, Table 5 also reveals factors with positive associations with the perception of Indigenous rights: gender (especially females), geographic location (residents of Quebec), residential zones (particularly urban cores), and the perceived importance of price and origin.

Finally, the results in Table 5 also demonstrate significant negative associations between the perception of a price increase and dietary preferences, specifically lacto-ovo vegetarian and vegetarian preferences. In contrast, respondents’ purchase history and the perceived importance of price and origin exhibit significant positive associations with the perception of a price increase.

### 4.5. Purchasing Behaviors

Please note that the three $R^{2}$ values are not relatively high; therefore, it is reasonable to include these three perceptions, socio-demographic variables (D1–D9 and Q1), as well as transformed variables $\tilde{Q13}$, $\tilde{Q14}$, and $\tilde{Q34}$ into the CLMs with purchasing behavior as the outcome variable. The primary objective is to elucidate the intricate relationship between consumer perceptions and actual purchasing decisions and to evaluate the extent to which concerns related to perceptions of environmental sustainability, economic considerations, Indigenous rights, and a price increase associated with consumer choices. Seven purchasing behaviors (Q5, Q7, Q8, Q9, Q11, Q17, Q21) are selected as response outcomes. The ensuing results are presented in Table 6.

The findings presented in Table 6 highlight the negative associations of pescatarian and vegetarian dietary preferences, as well as gender (particularly female), with the behavior of selecting fresh (never been frozen) salmon over frozen salmon. Conversely, the perception of economic considerations, geographical location (specifically respondents residing in Quebec), residential zones (particularly those in urban cores), respondents’ purchase history, the perceived importance of origin, and the perception of a price increase demonstrate significantly positive associations with the behavior of choosing fresh (never been frozen) salmon over frozen salmon. It should be noted that a unit increase in the perception of economic considerations corresponds to a 0.426 increase in such purchase behavior, and a unit increase in the perception of a price increase corresponds to a 0.200 increase.

The findings in Table 6 underscore the negative associations of lacto-ovo vegetarian, vegan, and vegetarian dietary preferences, as well as income (particularly in the range of $35,000 and $49,999), with the behavior of purchasing Canadian-sourced salmon. Conversely, the perception of economic considerations, age (particularly within the range preceding 1979), respondents’ purchase history, the perceived importance of origin, and the perception of a price increase demonstrate significantly positive associations with the behavior of purchasing Canadian-sourced salmon. Notably, a unit increase in the perception of economic considerations corresponds to a 0.759 increase in such purchase behavior, and a unit increase in the perception of a price increase corresponds to a 0.275 increase.

The findings in Table 6 unveil the negative associations of flexitarian, vegetarian, and other dietary preferences, as well as residential zones (particularly those in urban cores), with the behavior of selecting Canadian farm-raised salmon over salmon from another country. Conversely, the perception of environmental sustainability, the perception of economic considerations, age (particularly within the range preceding 1979), income (particularly in the $100,000 and $149,999 bracket), the perceived importance of origin, and the perception of a price increase demonstrate significantly positive associations with the behavior of purchasing Canadian farm-raised salmon over salmon from another country. Specifically, a unit increase in the perception of environmental sustainability corresponds to a 0.432 increase in such purchase behavior, a unit increase in the perception of economic considerations is linked to a 0.612 increase, and a unit increase in the perception of a price increase is associated with a 0.270 increase.

The findings in Table 6 reveal the positive associations of the perception of environmental sustainability, the perception of economic considerations, the perception of Indigenous rights, and education (especially with a college, CEGEP, or other non-university certificate or diploma, and high school diploma or equivalent) with the behavior of choosing Canadian farm-raised salmon over foreign wild salmon. In particular, a unit increase in the perception of environmental sustainability is associated with a 0.710 increment in such purchase behavior. Similarly, a unit increase in the perception of economic considerations corresponds to a 0.769 increase, and a unit increase in the perception of Indigenous rights is linked to a 0.227 increase in such purchase behavior.

The findings in Table 6 indicate the negative associations of the perception of environmental sustainability, flexitarian dietary preference, as well as education (particularly those with a high school diploma or equivalent), with the behavior of purchasing more Canadian farm-raised salmon to support Indigenous communities. Conversely, the perception of Indigenous rights demonstrates significantly positive associations with the behavior of buying more Canadian farm-raised salmon to support Indigenous communities. Notably, a unit increase in the perception of environmental sustainability corresponds to a 0.357 decrease in such purchase behavior, while a unit increase in the perception of a price increase is linked to a 6.487 increase.

The findings in Table 6 reveal the negative associations of the perception of economic considerations and age (especially within the range of the 1946–1964 bracket) with the behavior of purchasing more Canadian farm-raised salmon from farms that support Indigenous communities. Conversely, the perception of Indigenous rights demonstrates significantly positive associations with the behavior of buying more Canadian farm-raised salmon from farms that support Indigenous communities. Moreover, a unit increase in the perception of economic considerations corresponds to a 0.551 decrease in such purchase behavior, while a unit increase in the perception of a price increase is associated with a 7.915 increase.

Finally, the findings presented in Table 6 elucidate the negative associations of marital status (especially married or common-law), education (excluding the categorization ‘some high school’), the perceived importance of price, and the perception of a price increase with the behavior of willingness to pay more for salmon with a sustainable certification label. In contrast, the perception of environmental sustainability, Indigenous rights, pescatarian dietary preference, gender (especially female), income (those exceeding $100,000), residential zones (particularly those in urban cores), and the perceived importance of origin demonstrate significantly positive associations with the behavior of paying more for salmon with a sustainable certification label. Additionally, a unit increase in the perception of environmental sustainability corresponds to a 0.471 increase in such purchase behavior, a unit increase in the perception of Indigenous rights is linked to a 0.889 increase, while a unit increase in the perception of a price increase is associated with a 0.285 decrease.

## 5. Discussion

Table 1 and Table 2 present descriptive statistics derived from our survey data. Notably, responses to the Likert scale questions (Q2 to Q35) predominantly indicate neutrality or agreement, with over 76% of choices falling into these categories (neutral, agree, and strongly agree). Q4 (enjoyment of eating salmon regularly) shows a slight deviation, with approximately 69% falling into those categories.

Figure 1 illustrates the flow chart. Figure 2, Figure 3 and Figure 4 depict the process of dimensionality tests for the perceptions of environmental sustainability, economic considerations, and Indigenous rights, respectively. Each figure confirms a unidimensional characteristic in the related sub-domain, facilitating subsequent OLS or CLM analyses. Figure 5, Figure 6 and Figure 7 illustrate the graded response functions for each perception. Table 3 presents the results of reliability tests conducted on the entire survey and its three sub-domains, with both estimated values and 95% confidence intervals above the threshold of 0.7, indicating high consistency. Table 4 provides descriptive statistics of MAP-estimated latent traits.

In the assessment of the four perceptions, as demonstrated in Table 5, certain patterns emerge. Regarding environmental sustainability, statistically significant negative associations are observed with dietary preferences, gender, age, education, and geographic location, while positive correlations are found with household size, residential zones, and the perceived importance of price and origin. Similarly, economic considerations reflect a parallel pattern, displaying comparable negative associations with the aforementioned factors, while demonstrating positive associations with household size, geographic location, residential zones, and the perceived importance of price and origin. In the context of Indigenous rights, analogous negative associations are observed with environmental sustainability, alongside positive associations with gender, geographic location, residential zones, and the perceived importance of price and origin. Concerning the perception of a price increase, negative associations are apparent with dietary preferences, whereas positive correlations are established with respondents’ purchase history and the perceived importance of price and origin.

In the analysis of consumer purchasing behaviors highlighted in Table 6, particularly in the choice between fresh and frozen salmon (Q5), this study reveals negative effects associated with specific dietary preferences and female gender. Conversely, positive determinants include economic considerations, a price increase, Quebec residence, urban residence, purchase history, and the perceived importance of origin. Concerning the choice of Canadian-sourced salmon (Q7), negative associations are linked to certain dietary preferences and income, while positive associations are observed in economic considerations, a price increase, age, purchase history, and the perceived importance of origin. Negative associations with the selection of Canadian farm-raised salmon over those from another country (Q8) involve specific dietary preferences and urban residence, while positive associations are observed including environmental sustainability, economic considerations, a price increase, age, higher income, and the perceived importance of origin. Notably, the deduction drawn from these two purchasing behaviors (Q7 and Q8) implies that Canadians demonstrate a preference for Canadian-sourced farmed salmon compared to products from other countries, as shown by the observed positive significant association of the perceived importance of origin. Opting for Canadian farm-raised over foreign wild salmon (Q9) is positively associated with environmental sustainability, economic considerations, Indigenous rights, and education. Negative associations with supporting Indigenous communities (Q11) stem from considerations of environmental sustainability, lower education, and specific dietary preferences, while positive association is observed in the perceptions of Indigenous rights. Purchasing from farms supporting Indigenous communities (Q17) incurs negative correlations with economic considerations and age, while a positive association is observed with perceptions of Indigenous rights. Lastly, the willingness to pay more for sustainably certified salmon (Q21) is negatively associated with marital status, education, perceived importance of price, and a price increase. Positive inclinations toward such behavior are associated with environmental sustainability, perceptions of Indigenous rights, specific dietary preferences, female gender, higher income, urban residence, and the perceived importance of origin. The quantitative impacts of perceptions of environmental sustainability, economic considerations, Indigenous rights, and a price increase on consumer purchasing behaviors have been systematically calculated.

Comparisons with previous references pose challenges due to differences in the study objects and chosen variables. Nonetheless, some similar research exists. For instance, in the work by Zheng et al. [17], they utilize an index called the consequentiality script to compare two groups. Within the consequential treatment, agreements with Alaska origin and Alaska wild-caught, household size, and income are positively associated with the behavior of willingness to pay for wild-caught salmon, which is similar to our results. Interestingly, our study also finds a positive association between willingness to pay more for labeled salmon (Q21) and urban residence. However, this variable was not observed in Zheng et al.’s study [17], which might be due to their data being collected from three top-tier cities in China.

The advantage of our proposed method (CLM) is that the Likert scale outcome shall be directly regressed with sub-scale factors and socio-demographic covariates such that the normality assumption for outcomes is no longer required compared with other methods such as SEM. The CLM results can be used not only for interpretation but also for direct prediction of Likert scale outcomes. Moreover, CLM is flexible in the sense that various link functions can be selected depending on goodness-of-fit purposes or other rationale, for example, a probit link function can be used if the Likert scale outcome is acknowledged to be generated by a normal latent variable (in our settings, the use of logit link means that the Likert scale outcome is categorized based on a logistic distributed latent variable). However, the limitation is that the current version of CLM cannot accommodate potential structural equation relationships. To address this limitation, certain maximization methods of numerical approximation of likelihood function involving integrals of latent variables, for example, the EM algorithm, are required in the future.

This study is not exempt from certain limitations. First, the recruitment of participants through convenience sampling may still introduce selection bias, as individuals who perceive an increase in the price of salmon might be more inclined to participate with the anticipation of potential policies addressing the rising prices. Second, the formulation of survey questions may still carry the potential for subconscious bias. For instance, the wording of Question 34, “The price of salmon and other fish products has increased” may yield different responses compared to a counterpart statement such as “The price of salmon and other fish products has NOT increased”. The subtle alteration in phrasing could influence participant responses and introduce unintended biases into the data collection process.

Other contributions may further enrich the scope of this study. For instance, Głu-chowski et al. [36] elucidate how the perception of sensory quality, including sensory profile and consumer liking, correlates with consumer purchasing behaviors regarding salmon. In the future, by incorporating the sensory quality of Canadian salmon into our study framework, we may gain a more comprehensive understanding of these purchasing behaviors.

## 6. Conclusions

Based on the results of this study, several mathematical, managerial, and policy implications can be drawn, highlighting the significance of understanding perceptions related to environmental sustainability, economic considerations, Indigenous rights, and price increases in association with consumer purchasing behaviors.

From a mathematical perspective, this study contributes significantly to the existing body of knowledge by incorporating the perception of Indigenous rights. The identification of both positive and negative associations between perceptions and purchasing behaviors provides valuable insights into the complexity of consumer decision-making, highlighting the imperative for a nuanced understanding of these dynamics. Specifically, economic considerations, price increase, and origin importance demonstrate positive associations with the selection of fresh salmon (Q5) and the purchase of Canadian-sourced salmon (Q7). Furthermore, environmental sustainability, economic considerations, price increase, and origin importance are positively correlated with the purchase of Canadian farm salmon over imports (Q8). Although Indigenous rights exhibit positive associations with Indigenous-related behaviors (Q11) and (Q17), they are also positively associated with the selection of Canadian farm salmon over wild imports (Q9), alongside positive relationships with environmental sustainability and economic considerations. Q11 also reveals a negative association between environmental sustainability in salmon farming and Indigenous rights, underscoring the need for stakeholders to enhance sustainability practices to better serve Indigenous rights. A similar trend is observed in Q17. Intriguingly, in the willingness to pay more (Q21), environmental sustainability, Indigenous rights, price increases, and origin and price importance all contribute positively. From Q9 and Q21, it is evident that the perception of Indigenous rights is associated with purchase behavior, a facet not extensively explored in previous research. Moreover, a range of methodologies is employed in this study. Parallel analysis is utilized for dimensionality testing in each sub-domain, while the GRM and MAP techniques are applied to fit the targeted perceptions. Given the skewness observed in the behavioral variables, CLM is selected to analyze the associations between perceptions and purchase behaviors.

On a managerial level, the findings offer practical implications for businesses and marketers seeking to align their strategies with consumer preferences and values. By recognizing the significance of environmental sustainability, economic considerations, Indigenous rights, and price sensitivity, coupled with consumer motivation of origin in shaping purchasing behaviors, businesses can tailor their product offerings, marketing messages, and pricing strategies to resonate with consumers’ values and priorities. Illustratively, the perceptive recognition of Canadians’ preference for domestically sourced salmon and their inclination to choose Canadian farm-raised salmon over that of other countries broadens the scope of business opportunities within the realm of marketing. Additionally, understanding the differential impact of these perceptions across demographic segments enables firms to develop targeted marketing campaigns and product innovations that appeal to specific consumer segments, thereby enhancing competitiveness and market positioning.

From a policy standpoint, the results of this study underscore the importance of implementing regulatory frameworks and initiatives that promote sustainability, protect Indigenous rights, and address socio-economic disparities. Policies aimed at raising consumer awareness, promoting sustainable consumption practices, and ensuring transparency in supply chains can play a crucial role in influencing consumer behaviors and fostering a culture of responsible consumption. Furthermore, efforts to support Indigenous communities and promote fair trade practices can contribute to social equity and environmental stewardship, aligning with broader policy objectives related to sustainable development and social justice.

In conclusion, this study offers valuable insights into the complex interplay between perceptions and purchasing behaviors, highlighting the mathematical, managerial, and policy implications for understanding and influencing consumer decision-making processes. By addressing the underlying factors driving consumer preferences and values, businesses, policymakers, and stakeholders can work toward fostering more sustainable and ethical consumption patterns, thereby contributing to broader societal and environmental goals.

## Figures and Tables

**Table 1:** Frequencies (percentage) for socio-demographics.

Variables	Levels	Frequency
Dietary Preferences (D1)	Consumer with no dietary preferences	867 (80.43%)
	Consumer with specific religious or cultural dietary preferences	22 (2.04%)
	Flexitarian (vegetarian who occasionally eats meat and fish)	54 (5.01%)
	Lacto-ovo vegetarian (diet free of animal flesh but eats eggs and milk products)	14 (1.30%)
	Pescatarian (diet free of land animal flesh but eats eggs, fish, and milk products)	25 (2.32%)
	Vegan (diet free of all animal-based products)	17 (1.58%)
	Vegetarian (diet free of meat, fish, and fowl flesh)	16 (1.48%)
	Other ^†^	63 (5.84%)
Gender (D2)	Male	515 (47.77%)
	Female	538 (49.91%)
	Non-binary/third gender	18 (1.67%)
	Prefer not to say	7 (0.65%)
Marital Status (D3)	Divorced, separated, or widowed	127 (11.78%)
	Married or common-law	730 (67.72%)
	Single	221 (20.50%)
Age (D4)	Before 1946	38 (3.53%)
	From 1946 to 1964	347 (32.19%)
	From 1965 to 1979	206 (19.11%)
	From 1980 to 1994	366 (33.95%)
	After 1994	121 (11.22%)
Household Size (D5)	None	727 (67.44%)
	One	171 (15.86%)
	Two	137 (12.71%)
	Three or more	43 (3.99%)
Education (D6)	Advanced University Degree (Graduate)	197 (18.27%)
	College, CEGEP, or Other Non-University Certificate or Diploma	276 (25.60%)
	High School Diploma or Equivalent	129 (11.97%)
	Registered Apprenticeship or Other Trades Certificate or Diploma	82 (7.61%)
	Some High School	19 (1.76%)
	University Degree, Certificate, or Diploma	375 (34.79%)
Geographic Location (D7)	Atlantic Canada	77 (7.14%)
	British Columbia	156 (14.47%)
	Northern Region	3 (0.28%)
	Ontario	396 (36.73%)
	Prairies	186 (17.25%)
	Quebec	260 (24.12%)
Income (D8)	Less than $35,000	103 (9.55%)
	Between $35,000 and $49.99	97 (9.00%)
	Between $50,000 and $74,999	161 (14.94%)
	Between $75,000 and $99,999	185 (17.16%)
	Between $100,000 and $149,999	297 (27.55%)
	More than $150,000	235 (21.80%)
Residential Zone (D9)	Suburban	392 (36.36%)
	Small town, community, or rural	283 (26.25%)
	Urban Core	403 (37.38%)
Purchase History (Q1)	No	169 (15.68%)
	Yes	909 (84.32%)

Note: ^†^ The category ‘Raw foodist (a diet consisting mainly of raw fruits, vegetables, legumes, sprouts, and nuts)’ has been consolidated with the ‘Other’ category due to the presence of only one observation in the former.

**Table 2:** Frequencies (percentage) for variables from Question 2 to Question 35.

Variables	Names	Strongly Disagree	Disagree	Neutral	Agree	Strongly Agree
Q2	I enjoy eating salmon	118 (10.95%)	66 (6.12%)	141 (13.08%)	353 (32.75%)	400 (37.11%)
Q3	I enjoy eating salmon on special occasions	113 (10.48%)	94 (8.72%)	275 (25.51%)	370 (34.32%)	226 (20.96%)
Q4	I enjoy eating salmon regularly	143 (13.27%)	191 (17.72%)	228 (21.15%)	353 (32.75%)	163 (15.12%)
Q5	I prefer purchasing fresh (never been frozen) salmon over frozen salmon	67 (6.22%)	108 (10.02%)	357 (33.12%)	302 (28.01%)	244 (22.63%)
Q6	I enjoy eating salmon because it is a healthy choice	71 (6.59%)	55 (5.10%)	187 (17.35%)	513 (47.59%)	252 (23.38%)
Q7	I want to buy salmon that comes from Canada	48 (4.45%)	15 (1.39%)	169 (15.68%)	416 (38.59%)	430 (39.89%)
Q8	If I had the choice between farm-raised salmon from Canada or another country, I would buy Canadian salmon	37 (3.43%)	33 (3.06%)	173 (16.05%)	411 (38.13%)	424 (39.33%)
Q9	If I had a choice between buying wild salmon from a foreign country or farm-raised salmon from Canada, I would buy the Canadian salmon	101 (9.37%)	147 (13.64%)	252 (23.38%)	303 (28.11%)	275 (25.51%)
Q10	Most of the fresh (never been frozen) salmon in stores is from salmon farms	16 (1.48%)	33 (3.06%)	539 (50.00%)	397 (36.83%)	93 (8.63%)
Q11	I would purchase more Canadian farm-raised salmon if this supported Indigenous communities in Canada	116 (10.76%)	94 (8.72%)	416 (38.59%)	291 (26.99%)	161 (14.94%)
Q12	100% of current farmed salmon in BC is supported and overseen by local First Nations. This makes me more likely to buy BC-farmed salmon	94 (8.72%)	102 (9.46%)	420 (38.96%)	318 (29.50%)	144 (13.36%)
Q13	Price is an important factor when purchasing salmon products	25 (2.32%)	60 (5.57%)	193 (17.90%)	470 (43.60%)	330 (30.61%)
Q14	When I buy salmon, it is important for me to know where it comes from	23 (2.13%)	52 (4.82%)	195 (18.09%)	496 (46.01%)	312 (28.94%)
Q15	I prefer Atlantic salmon over Pacific salmon	64 (5.94%)	129 (11.97%)	663 (61.50%)	138 (12.80%)	84 (7.79%)
Q16	I prefer wild Atlantic salmon to farm-raised Atlantic salmon	17 (1.58%)	35 (3.25%)	508 (47.12%)	323 (29.96%)	195 (18.09%)
Q17	I would purchase more Canadian farm-raised salmon from farms that have local Indigenous community support and oversight	111 (10.30%)	97 (9.00%)	447 (41.47%)	308 (28.57%)	115 (10.67%)
Q18	I enjoy eating salmon because it is a more sustainable protein than other options	76 (7.05%)	102 (9.46%)	516 (47.87%)	293 (27.18%)	91 (8.44%)
Q19	Marine resource management and sustainability practices are very important for Canada’s salmon farming sector	19 (1.76%)	19 (1.76%)	216 (20.04%)	445 (41.28%)	379 (35.16%)
Q20	Canadian salmon farms are improving their environmental sustainability	43 (3.99%)	62 (5.75%)	642 (59.55%)	285 (26.44%)	46 (4.27%)
Q21	I would pay more for salmon with a sustainable certification label	80 (7.42%)	181 (16.79%)	385 (35.71%)	337 (31.26%)	95 (8.81%)
Q22	I believe sustainable-certified salmon farming is the future of salmon production in Canada	63 (5.84%)	65 (6.03%)	431 (39.98%)	402 (37.29%)	117 (10.85%)
Q23	Canadian farm-raised salmon has a lower carbon footprint than imported salmon	36 (3.34%)	34 (3.15%)	543 (50.37%)	354 (32.84%)	111 (10.30%)
Q24	I believe that Indigenous community oversight over salmon farms will help improve their sustainability	74 (6.86%)	111 (10.30%)	432 (40.07%)	334 (30.98%)	127 (11.78%)
Q25	I believe salmon farms support wild salmon stock recovery because they reduce pressure on the wild stocks	60 (5.57%)	85 (7.88%)	385 (35.71%)	442 (41.00%)	106 (9.83%)
Q26	I believe that the benefits of salmon farms are greater than any risks	91 (8.44%)	133 (12.34%)	516 (47.87%)	283 (26.25%)	55 (5.10%)
Q27	I believe that salmon can be responsibly farmed in the ocean	79 (7.33%)	109 (10.11%)	361 (33.49%)	445 (41.28%)	84 (7.79%)
Q28	I think the salmon farming sector is heavily regulated	45 (4.17%)	150 (13.91%)	626 (58.07%)	221 (20.50%)	36 (3.34%)
Q29	I like the idea of supporting coastal and Indigenous communities by purchasing Canadian farm-raised salmon	69 (6.40%)	60 (5.57%)	304 (28.20%)	470 (43.60%)	175 (16.23%)
Q30	I have confidence in the quality and welfare of salmon from Canada because of the oversight of the regulatory framework	54 (5.01%)	111 (10.30%)	418 (38.78%)	411 (38.13%)	84 (7.79%)
Q31	Supporting Canada’s youngest food production workforce in salmon farming is important to me	48 (4.45%)	80 (7.42%)	477 (44.25%)	379 (35.16%)	94 (8.72%)
Q32	I think Canada should produce more salmon to benefit Canadian consumers	45 (4.17%)	49 (4.55%)	321 (29.78%)	489 (45.36%)	174 (16.14%)
Q33	Canada exports most of its salmon	13 (1.21%)	44 (4.08%)	834 (77.37%)	151 (14.01%)	36 (3.34%)
Q34	The price of salmon and other fish products has increased	5 (0.46%)	9 (0.83%)	137 (12.71%)	523 (48.52%)	404 (37.48%)
Q35	I believe that reducing the BC farm-raised salmon supply in the North American market will negatively impact the retail price	16 (1.48%)	54 (5.01%)	450 (41.74%)	413 (38.31%)	145 (13.45%)

**Table 3:** Cronbach Alpha Reliability Test.

Sub-Domain	Variables	Cronbach Alpha	95% Confidence Boundaries	
-	D1 to Q35	0.921	Feldt	(0.914, 0.928)
			Duhachek	(0.915, 0.928)
Environmental sustainability	Q19–Q20, Q22–Q27	0.850	Feldt	(0.836, 0.863)
			Duhachek	(0.836, 0.863)
Economic considerations	Q28–Q33, Q35	0.795	Feldt	(0.776, 0.813)
			Duhachek	(0.778, 0.813)
Indigenous Rights	Q11–Q12, Q17, Q24, Q29	0.923	Feldt	(0.916, 0.930)
			Duhachek	(0.916, 0.930)

**Table 4:** Descriptive statistics for the MAP-estimated unidimensional latent traits.

Sub-Domain	Minimal	1st Quartile	Median	Mean	3rd Quartile	Maximal	Standard Deviation
Environmental sustainability	−3.11	−0.52	0.05	−0.00	0.56	2.70	0.94
Economic considerations	−3.21	−0.54	0.02	−0.00	0.57	2.71	0.92
Indigenous rights	−2.27	−0.52	−0.09	−0.00	0.66	2.01	0.96

**Table 5:** Results for four perceptions.

	Variables	Levels	Perception Outcomes			
			Environmental Sustainability	Economic Considerations	Indigenous Rights	Price Increase
Method			OLS	OLS	OLS	Logistic
Socio-demographic	D1 (Dietary Preferences)	Consumer with no dietary preferences Consumer with no dietary preferences	Reference			
		Consumer with specific religious or cultural dietary preferences	−0.163	−0.103	−0.021	−0.648
			(0.385)	(0.564)	(0.913)	(0.334)
		Flexitarian (vegetarian who occasionally eats meat and fish)	−0.303 *	−0.226 *	−0.088	−0.563
			(0.012)	(0.050)	(0.487)	(0.201)
		Lacto-ovo vegetarian (diet free of animal flesh but eats eggs and milk products)	−0.051	−0.009	0.112	−2.268 **
			(0.828)	(0.968)	(0.650)	(0.001)
		Pescatarian (diet free of land animal flesh but eats eggs, fish, and milk products)	0.035	0.054	0.264	−1.049
			(0.841)	(0.748)	(0.151)	(0.067)
		Vegan (diet free of all animal-based products)	−1.357 ***	−1.262 ***	−0.884 ***	−1.162
			(0.000)	(0.000)	(0.000)	(0.052)
		Vegetarian (diet free of meat, fish, and fowl flesh)	−0.314	−0.546 **	−0.080	−1.219 *
			(0.151)	(0.009)	(0.726)	(0.047)
		Other	−0.349 **	−0.354 ***	−0.238 *	−0.255
			(0.002)	(0.001)	(0.044)	(0.590)
	D2 (Gender)	Male	Reference			
		Female	−0.052	−0.058	0.172 **	0.329
			(0.334)	(0.266)	(0.003)	(0.124)
		Non-binary/third gender	−0.114	−0.550 **	0.266	−0.363
			(0.591)	(0.007)	(0.234)	(0.625)
		Prefer not to say	−0.964 **	−0.907 **	−0.725 *	−0.775
			(0.003)	(0.004)	(0.034)	(0.520)
	D3 (Marital Status)	Divorced, separated, or widowed	Reference			
		Married or common-law	0.091	0.098	0.112	−0.114
			(0.312)	(0.250)	(0.235)	(0.762)
		Single	−0.004	−0.078	0.113	−0.260
			(0.973)	(0.434)	(0.303)	(0.539)
	D4 (Age)	After 1994	Reference			
		Before 1946	−0.469 **	−0.103	−0.418 *	−0.060
			(0.006)	(0.524)	(0.019)	(0.944)
		From 1946 to 1964	−0.311 **	−0.053	−0.382 ***	−0.387
			(0.002)	(0.585)	(0.000)	(0.342)
		From 1965 to 1979	−0.358 ***	−0.207*	−0.242 *	−0.460
			(0.001)	(0.037)	(0.027)	(0.258)
		From 1980 to 1994	−0.203 *	−0.153	−0.121	−0.384
			(0.032)	(0.091)	(0.224)	(0.301)
	D5 (Household Size)	None	Reference			
		One	0.186 *	0.208 **	0.083	−0.461
			(0.018)	(0.005)	(0.313)	(0.127)
		Two	0.127	0.206 *	−0.067	−0.046
			(0.138)	(0.012)	(0.350)	(0.891)
		Three or more	−0.205	−0.084	0.084	−0.835
			(0.140)	(0.527)	(0.645)	(0.064)
	D6 (Education)	Advanced University Degree (Graduate)	Reference			
		College, CEGEP, or Other Non-University Certificate or Diploma	−0.058	−0.062	−0.152	0.577
			(0.491)	(0.444)	(0.088)	(0.084)
		High School Diploma or Equivalent	−0.038	−0.044	−0.120	0.450
			(0.705)	(0.645)	(0.257)	(0.247)
		Registered Apprenticeship or Other Trades Certificate or Diploma	−0.450 ***	−0.349 **	−0.395 **	0.363
			(0.000)	(0.002)	(0.001)	(0.441)
		Some High School	0.085	−0.163	−0.218	0.459
			(0.690)	(0.423)	(0.331)	(0.537)
		University Degree, Certificate, or Diploma	0.137	0.074	0.023	0.198
			(0.072)	(0.309)	(0.773)	(0.483)
	D7 (Geographic Location)	Atlantic Canada	Reference			
		British Columbia	−0.438 ***	−0.370 **	−0.261 *	−0.747
			(0.000)	(0.001)	(0.038)	(0.186)
		Northern Region	−0.450	0.150	0.253	−1.241
			(0.374)	(0.755)	(0.633)	(0.406)
		Ontario	−0.043	0.004	0.174	−0.615
			(0.689)	(0.972)	(0.120)	(0.236)
		Prairies	−0.053	−0.105	0.068	−0.786
			(0.646)	(0.342)	(0.578)	(0.147)
		Quebec	0.216	0.327 **	0.355 **	−0.538
			(0.053)	(0.002)	(0.002)	(0.318)
	D8 (Income)	Less than $35,000	Reference			
		Between $35,000 and $49,999	−0.076	−0.053	0.062	0.099
			(0.540)	(0.654)	(0.630)	(0.829)
		Between $50,000 and $74,999	0.057	0.020	0.066	0.811
			(0.609)	(0.850)	(0.571)	(0.078)
		Between $75,000 and $99,999	−0.133	−0.161	−0.133	0.054
			(0.240)	(0.136)	(0.260)	(0.901)
		Between $100,000 and $149,999	−0.108	−0.087	−0.115	0.161
			(0.328)	(0.405)	(0.318)	(0.702)
		More than $150,000	−0.139	−0.161	−0.220	0.199
			(0.238)	(0.151)	(0.074)	(0.656)
	D9 (Residential Zone)	Suburban	Reference			
		Small town, community, or rural	0.143 *	0.105	0.068	0.128
			(0.036)	(0.104)	(0.340)	(0.643)
		Urban Core	0.114	0.129 *	0.205 **	0.042
			(0.068)	(0.031)	(0.002)	(0.861)
	Q1 (Purchase History)	No	Reference			
		Yes	0.099	0.181 *	0.074	1.229 ***
			(0.207)	(0.016)	(0.366)	(0.000)
	$\tilde{Q13}$ (Perceived importance of price)	No	Reference			
		Yes	0.433 ***	0.478 ***	0.350 ***	1.050 ***
			(0.000)	(0.000)	(0.000)	(0.000)
	$\tilde{Q14}$ (Perceived importance of origin)	No	Reference			
		Yes	0.151 *	0.194 **	0.339 ***	1.141 ***
			(0.019)	(0.002)	(0.000)	(0.000)
	Adjusted R^2^		0.1932	0.2325	0.1595	-

Notes: * *p* < 0.05, ** *p* < 0.01, *** *p* < 0.001.

**Table 6:** Results for purchasing behaviors using the cumulative link model.

	Variables	Levels	Purchasing Behaviors						
			Q5 (Selection of Fresh (Never Been Frozen) Salmon over Frozen Salmon)	Q7 (Purchase Canadian-Sourced Salmon)	Q8 (Selection of Canadian Farm-Raised Salmon over Salmon from Another Country)	Q9 (Selection of Canadian Farm-Raised Salmon over Foreign Wild Salmon)	Q11 (Purchase more Canadian Farm-Raised Salmon to Support Indigenous Communities)	Q17 (Purchase more Canadian Farm-Raised Salmon from farms that Support Indigenous Communities)	Q21 (Willingness to Pay More for Salmon with a Sustainable Certification Label)
	Environmental Sustainability		−0.211	−0.216	0.432 ***	0.710 ***	−0.357 *	−0.114	0.471 ***
			(0.050)	(0.066)	(0.000)	(0.000)	(0.028)	(0.524)	(0.000)
	Economic Considerations		0.426 ***	0.759 ***	0.612 ***	0.769 ***	−0.229	−0.551 **	−0.015
			(0.000)	(0.000)	(0.000)	(0.000)	(0.168)	(0.003)	(0.901)
	Indigenous Rights		0.062	0.157	0.163	0.227 *	6.487 ***	7.915 ***	0.889 ***
			(0.462)	(0.090)	(0.074)	(0.011)	(0.000)	(0.000)	(0.000)
Socio-demographic	D1 (Dietary Preferences)	Consumer with no dietary preferences	Reference						
		Consumer with specific religious or cultural dietary preferences	0.317	0.297	−0.212	−0.659	−0.453	0.471	0.020
			(0.452)	(0.503)	(0.595)	(0.105)	(0.476)	(0.548)	(0.962)
		Flexitarian (vegetarian who occasionally eats meat and fish)	0.222	−0.007	−0.614 *	−0.041	−0.757 *	−0.007	0.295
			(0.382)	(0.981)	(0.029)	(0.879)	(0.036)	(0.987)	(0.281)
		Lacto-ovo vegetarian (diet free of animal flesh but eats eggs and milk products)	0.200	−1.341 *	−0.061	0.061	−0.368	−0.043	0.144
			(0.688)	(0.012)	(0.905)	(0.899)	(0.642)	(0.965)	(0.763)
		Pescatarian (diet free of land animal flesh but eats eggs, fish, and milk products)	−1.071 **	0.037	0.797	−0.255	0.506	0.592	1.113 **
			(0.006)	(0.931)	(0.062)	(0.496)	(0.353)	(0.365)	(0.005)
		Vegan (diet free of all animal-based products)	−0.776	−1.089 *	0.035	0.635	1.358	−1.653	−0.107
			(0.122)	(0.036)	(0.943)	(0.215)	(0.078)	(0.169)	(0.840)
		Vegetarian (diet free of meat, fish, and fowl flesh)	−1.236 *	−1.559 **	−0.920 *	−0.110	−0.345	1.093	0.911
			(0.012)	(0.002)	(0.049)	(0.812)	(0.603)	(0.133)	(0.052)
		Other	−0.085	0.015	−0.628 *	−0.383	−0.116	0.237	0.197
			(0.733)	(0.955)	(0.020)	(0.127)	(0.733)	(0.542)	(0.439)
	D2 (Gender)	Male	Reference						
		Female	−0.441 ***	0.035	−0.079	0.056	−0.105	−0.071	0.373 **
			(0.000)	(0.786)	(0.718)	(0.648)	(0.544)	(0.719)	(0.002)
		Non-binary/third gender	−0.415	−0.344	−0.449	−0.232	−1.125	−0.588	0.095
			(0.354)	(0.492)	(0.345)	(0.619)	(0.079)	(0.432)	(0.838)
		Prefer not to say	0.125	0.192	0.218	0.991	−0.890	1.562	1.215
			(0.850)	(0.795)	(0.769)	(0.165)	(0.326)	(0.148)	(0.112)
	D3 (Marital Status)	Divorced, separated, or widowed	Reference						
		Married or common-law	−0.366	−0.213	−0.093	−0.007	−0.415	0.238	−0.446 *
			(0.064)	(0.323)	(0.660)	(0.973)	(0.143)	(0.455)	(0.024)
		Single	−0.254	−0.043	−0.097	−0.072	−0.287	−0.183	−0.397
			(0.270)	(0.864)	(0.695)	(0.759)	(0.390)	(0.628)	(0.089)
	D4 (Age)	After 1994	Reference						
		Before 1946	−0.054	1.088 **	0.953 *	0.222	−0.432	−0.705	0.097
			(0.887)	(0.009)	(0.021)	(0.550)	(0.416)	(0.222)	(0.793)
		From 1946 to 1964	−0.275	0.707 **	0.701 **	0.327	0.172	−1.103 ***	0.420
			(0.215)	(0.003)	(0.003)	(0.143)	(0.605)	(0.004)	(0.063)
		From 1965 to 1979	−0.016	0.722 **	0.643 **	0.253	−0.476	−0.511	0.108
			(0.944)	(0.003)	(0.007)	(0.274)	(0.167)	(0.198)	(0.644)
		From 1980 to 1994	−0.009	0.362	0.354	0.242	−0.537	−0.313	0.184
			(0.966)	(0.102)	(0.097)	(0.237)	(0.084)	(0.384)	(0.385)
	D5 (Household Size)	None	Reference						
		One	0.004	−0.173	0.064	0.172	0.212	0.078	0.068
			(0.982)	(0.348)	(0.726)	(0.326)	(0.388)	(0.787)	(0.697)
		Two	0.031	−0.125	−0.090	−0.185	0.213	−0.192	0.236
			(0.866)	(0.534)	(0.648)	(0.321)	(0.433)	(0.532)	(0.437)
		Three or more	−0.352	0.086	0.042	−0.068	0.445	−0.297	0.294
			(0.262)	(0.790)	(0.891)	(0.819)	(0.271)	(0.519)	(0.130)
	D6 (Education)	Advanced University Degree (Graduate)	Reference						
		College, CEGEP, or Other Non-University Certificate or Diploma	−0.069	−0.115	0.233	0.602 **	−0.357	−0.198	−0.526 **
			(0.714)	(0.561)	(0.240)	(0.001)	(0.172)	(0.509)	(0.005)
		High School Diploma or Equivalent	0.073	−0.390	0.115	0.576 *	−0.845 **	0.486	−0.658 **
			(0.743)	(0.100)	(0.627)	(0.010)	(0.008)	(0.185)	(0.004)
		Registered Apprenticeship or Other Trades Certificate or Diploma	0.294	0.158	0.336	0.311	−0.713	−0.289	−0.674 *
			(0.255)	(0.579)	(0.235)	(0.249)	(0.072)	(0.520)	(0.014)
		Some High School	0.067	−0.465	0.307	0.764	−0.070	−1.088	−0.188
			(0.895)	(0.374)	(0.540)	(0.129)	(0.918)	(0.151)	(0.685)
		University Degree, Certificate, or Diploma	0.019	−0.255	−0.165	0.197	−0.425	0.320	−0.424 *
			(0.909)	(0.150)	(0.349)	(0.233)	(0.067)	(0.232)	(0.012)
	D7 (Geographic Location)	Atlantic Canada	Reference						
		British Columbia	0.221	0.419	−0.006	−0.242	0.309	−0.414	0.103
			(0.405)	(0.151)	(0.982)	(0.367)	(0.412)	(0.341)	(0.702)
		Northern Region	−0.862	−0.790	−0.189	−1.892	−1.874	−1.895	0.619
			(0.384)	(0.449)	(0.847)	(0.091)	(0.164)	(0.360)	(0.552)
		Ontario	0.149	−0.167	−0.068	−0.338	−0.066	−0.306	−0.074
			(0.529)	(0.520)	(0.787)	(0.154)	(0.844)	(0.439)	(0.759)
		Prairies	0.144	0.183	0.167	−0.119	0.153	−0.553	−0.349
			(0.572)	(0.513)	(0.536)	(0.640)	(0.677)	(0.199)	(0.176)
		Quebec	0.608*	−0.295	0.348	0.210	0.075	−0.524	−0.007
			(0.015)	(0.279)	(0.193)	(0.404)	(0.832)	(0.214)	(0.977)
	D8 (Income)	Less than $35,000	Reference						
		Between $35,000 and $49,999	−0.324	−0.725 *	0.094	−0.201	0.290	−0.032	0.238
			(0.236)	(0.014)	(0.746)	(0.474)	(0.473)	(0.945)	(0.384)
		Between $50,000 and $74,999	−0.044	−0.476	0.099	−0.124	0.007	−0.269	0.453
			(0.857)	(0.078)	(0.706)	(0.627)	(0.984)	(0.517)	(0.069)
		Between $75,000 and $99,999	0.006	−0.397	0.185	−0.220	0.057	−0.408	0.261
			(0.981)	(0.148)	(0.484)	(0.394)	(0.878)	(0.328)	(0.298)
		Between $100,000 and $149,999	0.088	−0.231	0.543 *	0.080	0.177	−0.043	0.637 **
			(0.720)	(0.388)	(0.035)	(0.754)	(0.621)	(0.916)	(0.009)
		More than $150,000	0.096	−0.450	0.404	−0.279	0.066	−0.340	0.643 *
			(0.712)	(0.113)	(0.142)	(0.299)	(0.863)	(0.433)	(0.014)
	D9 (Residential Zone)	Suburban	Reference						
		Small town, community, or rural	0.108	0.173	−0.036	0.088	0.143	−0.288	0.260
			(0.467)	(0.287)	(0.821)	(0.562)	(0.503)	(0.242)	(0.087)
		Urban Core	0.313 *	−0.109	−0.341 *	−0.216	−0.195	−0.291	0.439 **
			(0.022)	(0.455)	(0.019)	(0.120)	(0.321)	(0.191)	(0.002)
	Q1 (Purchase History)	No	Reference						
		Yes	0.861 ***	1.175 ***	0.121	0.009	0.082	−0.186	−0.054
			(0.000)	(0.000)	(0.500)	(0.960)	(0.751)	(0.543)	(0.758)
	$\tilde{Q13}$ (Perceived importance of price)	No	Reference						
		Yes	0.043	−0.066	0.280	−0.043	0.263	−0.093	−0.694 ***
			(0.769)	(0.674)	(0.070)	(0.768)	(0.211)	(0.702)	(0.000)
	$\tilde{Q14}$ (Perceived importance of origin)	No	Reference						
		Yes	0.800 ***	1.874 ***	0.827 ***	−0.089	−0.135	0.401	1.122 ***
			(0.000)	(0.000)	(0.000)	(0.530)	(0.511)	(0.091)	(0.000)
	$\tilde{Q34}$ (Price increase)	No	Reference						
		Yes	0.200 *	0.275 **	0.270 **	0.044	−0.039	−0.205	−0.285 **
			(0.022)	(0.003)	(0.003)	(0.617)	(0.760)	(0.155)	(0.002)
Threshold coefficients	$\alpha_{1}$ (Strongly Disagree | Disagree)		−1.110 *	−1.139	−1.616 **	−2.692 ***	−8.511 ***	−11.622 ***	−3.805 ***
			(0.047)	(0.062)	(0.007)	(0.000)	(0.000)	(0.000)	(0.000)
	$\alpha_{2}$ (Disagree | Neither agree nor disagree)		0.104	−0.704	−0.798	−1.227 **	−5.855 ***	−8.032 ***	−2.073 ***
			(0.851)	(0.245)	(0.174)	(0.030)	(0.000)	(0.000)	(0.000)
	$\alpha_{3}$ (Neither agree nor disagree | Agree)		1.962 ***	1.549 *	1.074	0.209	0.398	0.212	−0.0195
			(0.000)	(0.010)	(0.065)	(0.709)	(0.633)	(0.824)	(0.973)
	$\alpha_{4}$ (Agree | Strongly Agree)		3.387 ***	3.954 ***	3.314 ***	1.818 **	5.427 ***	7.441 ***	2.472 ***
			(0.000)	(0.000)	(0.000)	(0.001)	(0.000)	(0.000)	(0.000)
	Log-likelihood		−1460.27	−1059.56	−1132.60	−1404.83	−565.47	−421.15	−1315.72

Notes: * *p* < 0.05, ** *p* < 0.01, *** *p* < 0.001

## Data Availability

Dataset available on request from the authors.

## Supplementary Materials

The following supporting information can be downloaded at: https://www.mdpi.com/article/10.3390/foods13091309/s1, File S1: Questionnaire—Consumer Trust & Salmon in Canada.
