# Supplementary material for: Consumer Perceptions of the Canadian Salmon Sector and Their Associations with Behaviors: A Perspective from Indigenous Rights
Source: Foods. 2024 Apr 24;13(9):1309. doi: 10.3390/foods13091309 (PMC11083706; doi:10.3390/foods13091309)
Supplement: Supplementary file 1 [file foods-13-01309-s001.zip › foods-2943776-supplementary.pdf]

File S1: Questionnaire – Consumer Trust & Salmon in Canada

C1 Clicking on the "Agree" button indicates that: You have read the above information- You voluntarily agree to participate- You are 18 years of age or older- You have been living in Canada for at least the last 12 months

o Agree (1)

o Disagree (2)

C2 Clicking on the "Agree" button indicates that:

- You have read the above information

o Agree (1)

o Disagree (2)

D1 As a consumer, I consider myself a...

o Consumer with no dietary preferences (1)

o Vegetarian (diet free of meat, fish, and fowl flesh) (2)

o Lacto-ovo vegetarian (diet free of animal flesh but eats eggs and milk products) (3)

o Pescatarian (diet free of land animal flesh but eats eggs, fish, and milk products) (4)

o Vegan (diet free of all animal-based products) (5)

o Raw foodist (diet consisting mainly of raw fruits, vegetables, legumes, sprouts and nuts) (6)

o Flexitarian (vegetarian who occasionally eats meat and fish) (7)

o Consumer with specific religious or cultural dietary preferences (8)

o Other (9) ___

D2 What is your gender?

o Man (1)

o Woman (2)

- o Other (3)

D3 What is your marital status?

- o Single (1)
- o Married or common-law (2)
- o Divorced, separated or widowed (3)

D4 Please select the age range which best describes you:

- o After 1994 (1)
- o From 1980 to 1994 (2)
- o From 1965 to 1979 (3)
- o From 1946 to 1964 (4)
- o Before 1946 (5)

D5 How many children live in your household?

- o One (1)
- o Two (2)
- o Three or more (3)
- o None (4)

D6 Please select the highest level of education which you have achieved:

- o Some High School (1)
- o High School Diploma or Equivalent (2)
- o Registered Apprenticeship or Other Trades Certificate or Diploma (3)
- o College, CEGEP or Other Non-University Certificate or Diploma (4)
- o University Degree, Certificate or Diploma (5)
- o Advanced University Degree (Graduate) (6)

D7 In which region do you currently reside?

- British Columbia (1)
- Prairies (2)
- Ontario (3)
- Quebec (4)
- Atlantic Canada (5)
- Northern Region (6)

D8 Which of the following categories best describes your total household income? That is, the total income of all the persons in your household combined before taxes.

- Less than $35,000 (1)
- Between $35,000 and $49,999 (2)
- Between $50,000 and $74,999 (3)
- Between $75,000 and $99,999 (4)
- Between $100,000 and $149,999 (5)
- $150,000 + (6)

D9 Which of the following best describes where you currently reside?

- Urban Core (1)
- Suburban (2)
- Small town. community or rural (3)

Salmon consumer

Q1 Have you purchased Canadian salmon products in the past?

- Yes (1)
- No (2)

Consumer motivation

Q2 I enjoy eating salmon

- Strongly disagree (1)

- o Disagree (2)
- o Neither agree nor disagree (3)
- o Agree (4)
- o Strongly agree (5)

Q3 I enjoy eating salmon on special occasions

- o Strongly disagree (1)
- o Disagree (2)
- o Neither agree nor disagree (3)
- o Agree (4)
- o Strongly agree (5)

Q4 I enjoy eating salmon regularly

- o Strongly disagree (1)
- o Disagree (2)
- o Neither agree nor disagree (3)
- o Agree (4)
- o Strongly agree (5)

Q5 I prefer purchasing fresh (never been frozen) salmon over frozen salmon

- o Strongly disagree (1)
- o Disagree (2)
- o Neither agree nor disagree (3)
- o Agree (4)
- o Strongly agree (5)

Q6 I enjoy eating salmon because it is a healthy choice

- o Strongly disagree (1)
- o Disagree (2)

- Neither agree nor disagree (3)
- Agree (4)
- Strongly agree (5)

Q7 I want to buy salmon that comes from Canada

- Strongly disagree (1)
- Disagree (2)
- Neither agree nor disagree (3)
- Agree (4)
- Strongly agree (5)

Q8 If I had the choice between farm-raised salmon from Canada or another country, I would buy Canadian salmon

- Strongly disagree (1)
- Disagree (2)
- Neither agree nor disagree (3)
- Agree (4)
- Strongly agree (5)

Q9 If I had a choice between buying wild salmon from a foreign country or farm-raised salmon from Canada, I would buy the Canadian salmon

- Strongly disagree (1)
- Disagree (2)
- Neither agree nor disagree (3)
- Agree (4)
- Strongly agree (5)

Q10 Most of the fresh (never been frozen) salmon in stores is from salmon farms

- Strongly disagree (1)
- Disagree (2)

- Neither agree nor disagree (3)
- Agree (4)
- Strongly agree (5)

Q11 I would purchase more Canadian farm-raised salmon if this supported Indigenous communities in Canada

- Strongly disagree (1)
- Disagree (2)
- Neither agree nor disagree (3)
- Agree (4)
- Strongly agree (5)

Q12 100% of current farmed salmon in BC is supported and overseen by local First Nations. This makes me more likely to buy BC farmed salmon

- Strongly disagree (1)
- Disagree (2)
- Neither agree nor disagree (3)
- Agree (4)
- Strongly agree (5)

Q13 Price is an important factor when purchasing salmon products

- Strongly disagree (1)
- Disagree (2)
- Neither agree nor disagree (3)
- Agree (4)
- Strongly agree (5)

Q14 When I buy salmon, it's important for me to know where it comes from

- Strongly disagree (1)
- Disagree (2)

- Neither agree nor disagree (3)
- Agree (4)
- Strongly agree (5)

Q15 I prefer Atlantic salmon over Pacific salmon

- Strongly disagree (1)
- Disagree (2)
- Neither agree nor disagree (3)
- Agree (4)
- Strongly agree (5)

Q16 I prefer wild Atlantic salmon to farm-raised Atlantic salmon

- Strongly disagree (1)
- Disagree (2)
- Neither agree nor disagree (3)
- Agree (4)
- Strongly agree (5)

Q17 I would purchase more Canadian farm-raised salmon from farms that have local Indigenous community support and oversight

- Strongly disagree (1)
- Disagree (2)
- Neither agree nor disagree (3)
- Agree (4)
- Strongly agree (5)

Sustainability

Q18 I enjoy eating salmon because it is a more sustainable protein than other options

- Strongly disagree (1)

- o Disagree (2)
- o Neither agree nor disagree (3)
- o Agree (4)
- o Strongly agree (5)

Q19 Marine resource management and sustainability practices are very important for Canada's salmon farming sector

- o Strongly disagree (1)
- o Disagree (2)
- o Neither agree nor disagree (3)
- o Agree (4)
- o Strongly agree (5)

Q20 Canadian salmon farms are improving their environmental sustainability

- o Strongly disagree (1)
- o Disagree (2)
- o Neither agree nor disagree (3)
- o Agree (4)
- o Strongly agree (5)

Q21 I would pay more for salmon with a sustainable certification label

- o Strongly disagree (1)
- o Disagree (2)
- o Neither agree nor disagree (3)
- o Agree (4)
- o Strongly agree (5)

Q22 I believe sustainable-certified salmon farming is the future of salmon production for Canada

- o Strongly disagree (1)

o Disagree (2)

o Neither agree nor disagree (3)

o Agree (4)

o Strongly agree (5)

Q23 Canadian farm-raised salmon has a lower carbon footprint than imported salmon

o Strongly disagree (1)

o Disagree (2)

o Neither agree nor disagree (3)

o Agree (4)

o Strongly agree (5)

Q24 I believe that Indigenous community oversight over salmon farms will help improve their sustainability

o Strongly disagree (1)

o Disagree (2)

o Neither agree nor disagree (3)

o Agree (4)

o Strongly agree (5)

Q25 I believe salmon farms support wild salmon stock recovery because they reduce pressure on the wild stocks

o Strongly disagree (1)

o Disagree (2)

o Neither agree nor disagree (3)

o Agree (4)

o Strongly agree (5)

Q26 I believe that the benefits of salmon farms are greater than any risks

o Strongly disagree (1)

o Disagree (2)

- o Neither agree nor disagree (3)
- o Agree (4)
- o Strongly agree (5)

Q27 I believe that salmon can be responsibly farmed in the ocean

- o Strongly disagree (1)
- o Disagree (2)
- o Neither agree nor disagree (3)
- o Agree (4)
- o Strongly agree (5)

Economic considerations

Q28 I think the salmon farming sector is heavily regulated

- o Strongly disagree (1)
- o Disagree (2)
- o Neither agree nor disagree (3)
- o Agree (4)
- o Strongly agree (5)

Q29 I like the idea of supporting coastal and Indigenous communities by purchasing Canadian farm-raised salmon

- o Strongly disagree (1)
- o Disagree (2)
- o Neither agree nor disagree (3)
- o Agree (4)
- o Strongly agree (5)

Q30 I have confidence in the quality and welfare of salmon from Canada because of the oversight of the regulatory framework

- o Strongly disagree (1)

- o Disagree (2)
- o Neither agree nor disagree (3)
- o Agree (4)
- o Strongly agree (5)

Q31 Supporting Canada's youngest food production workforce in salmon farming is important to me

- o Strongly disagree (1)
- o Disagree (2)
- o Neither agree nor disagree (3)
- o Agree (4)
- o Strongly agree (5)

Q32 I think Canada should produce more salmon to benefit Canadian consumers

- o Strongly disagree (1)
- o Disagree (2)
- o Neither agree nor disagree (3)
- o Agree (4)
- o Strongly agree (5)

Q33 Canada exports most of its salmon

- o Strongly disagree (1)
- o Disagree (2)
- o Neither agree nor disagree (3)
- o Agree (4)
- o Strongly agree (5)

Q34 The price of salmon and other fish products has increased

- o Strongly disagree (1)

o Disagree (2)

o Neither agree nor disagree (3)

o Agree (4)

o Strongly agree (5)

Q35 I believe that reducing the BC farm-raised salmon supply in the North American market will negatively impact the retail price

o Strongly disagree (1)

o Disagree (2)

o Neither agree nor disagree (3)

o Agree (4)

o Strongly agree (5)

Final question

F1 Is there anything else you would like to add regarding your current perceptions towards the Canadian salmon sector?

o Please type your answer in the space provided. (1)

____

o Nothing to add at this time (2)
